# Structure, gating and interactions of the voltage-dependent anion channel

**DOI:** 10.1007/s00249-021-01515-7

**Published:** 2021-03-29

**Authors:** Eszter E. Najbauer, Stefan Becker, Karin Giller, Markus Zweckstetter, Adam Lange, Claudia Steinem, Bert L. de Groot, Christian Griesinger, Loren B. Andreas

**Affiliations:** 1 Department of NMR-Based Structural Biology, Max Planck Institute for Biophysical Chemistry, Am Fassberg 11, 37077 Göttingen, Germany; 2 Senior Research Group of Translational Structural Biology in Dementia, Deutsches Zentrum Für Neurodegenerative Erkrankungen (DZNE), Von-Siebold-Str. 3a, 37075 Göttingen, Germany; 3 Department of Neurology, University Medical Center Göttingen, University of Göttingen, Waldweg 33, 37073 Göttingen, Germany; 4 Department of Molecular Biophysics, Leibniz-Forschungsinstitut Für Molekulare Pharmakologie, 13125 Berlin, Germany; 5 Institut Für Biologie, Humboldt-Universität Zu Berlin, 10115 Berlin, Germany; 6 Institute of Organic and Biomolecular Chemistry, University of Göttingen, Göttingen, Germany; 7 Max-Planck Institute for Dynamics and Self-Organization, Göttingen, Germany; 8 Department of Theoretical and Computational Biophysics, Max-Planck Institute for Biophysical Chemistry, Am Fassberg 11, 37077 Göttingen, Germany

**Keywords:** Voltage dependent anion channel, Solid-state NMR, Magic-angle spinning, Membrane protein, Electrophysiology, Molecular dynamics simulations

## Abstract

The voltage-dependent anion channel (VDAC) is one of the most highly abundant proteins found in the outer mitochondrial membrane, and was one of the earliest discovered. Here we review progress in understanding VDAC function with a focus on its structure, discussing various models proposed for voltage gating as well as potential drug targets to modulate the channel’s function. In addition, we explore the sensitivity of VDAC structure to variations in the membrane environment, comparing DMPC-only, DMPC with cholesterol, and near-native lipid compositions, and use magic-angle spinning NMR spectroscopy to locate cholesterol on the outside of the β-barrel. We find that the VDAC protein structure remains unchanged in different membrane compositions, including conditions with cholesterol.

## Introduction

Mitochondria play a central role in eukaryotic cells, and are involved in a plethora of functions, including energy production, regulation of metabolism, and participation in signaling pathways. Mitochondria are generally thought to be of endosymbiotic origin (Martin and Mentel 2010), which explains the existence of mitochondrial DNA and a delimiting membrane containing β-barrel integral membrane proteins. The inner- and outer mitochondrial membranes (IMM and OMM) separate the matrix of mitochondria from the cytosol, while the proteins and protein complexes embedded in these conduct oxidative phosphorylation, and ensure communication between the mitochondrion and the cell.

Cells are mostly powered by the ATP produced in mitochondria through oxidative phosphorylation (Alberts et al. 2002). In this process, electrons generated from NADH are passed along by a series of respiratory enzyme complexes located in the inner mitochondrial membrane, and with the resulting energy protons are pumped across the membrane. The arising proton gradient is used as an energy source by ATP synthase to produce ATP from ADP and phosphate (Saraste 1999). The continuous flow of ATP, ADP, small molecules, and proteins between mitochondria and the cytoplasm is ensured by integral membrane proteins in both the IMM and OMM. In the IMM, the ATP/ADP carrier (Duee and Vignais 1965; Pfaff et al. 1965) is responsible for the exchange of nucleotides, while in the OMM it is the voltage-dependent anion channel (VDAC), classified as a porin, which allows the exchange of metabolites and ions across the OMM. There are two other large protein complexes in the OMM, the sorting and assembly machinery (SAM) (Wiedemann et al. 2003) and mitochondrial distribution and morphology (Mdm) complex. In addition, a supercomplex spanning the intermembrane space and formed by translocases of the outer (TOM) and inner (TIM) membrane (Chacinska et al. 2003) mediates translocation of all synthesized proteins from the cytoplasm to the matrix of the mitochondrion.

The voltage-dependent anion channel (VDAC) is the most abundant protein in the outer mitochondrial membrane, covering up to 80% of membrane surface area in high-density regions (Goncalves et al. 2007). The protein was first described in 1976 when it was isolated from mitochondria and incorporated into lipid bilayers (Schein et al. 1976). In membranes, the ~ 30 kDa (Mannella 1982; Roos et al. 1982; Zalman et al. 1980) protein forms 2D crystalline arrays of aqueous pores about 3 nm in diameter (Benz 1994; Mannella 1982). VDAC is responsible for the permeability of the OMM to small molecules, and though the channels do not have a sharp exclusion limit, they are generally permeable to molecules up to 3–6 kDa in size (Benz 1994; Zalman et al. 1980).

VDACs are involved in a multitude of cellular functions. The channel is the main conduit for ATP and ADP flow between the cytosol and mitochondria and controls Ca^2+^ homeostasis in mitochondria. It has been suggested that VDAC oligomerization might lead to the formation of a mega-pore mediating the release of cytochrome *c* and other pro-apoptotic factors such as hexokinase, thus playing a key role in apoptosis (Ben-Hail and Shoshan-Barmatz 2016; Zalk et al. 2005). So far, however, there is no high resolution structural data proving the existence of such a species. Recently, based on VDAC’s interaction with mitoNEET, the channel’s involvement in ferroptosis has also been suggested (Lipper et al. 2019). As the binding partner of a plethora of proteins, VDAC has been implicated in various diseases, such as Alzheimer’s disease, Parkinsons’s disease and cancer (Caterino et al. 2017).

## Materials and methods

**Fig. 1:**
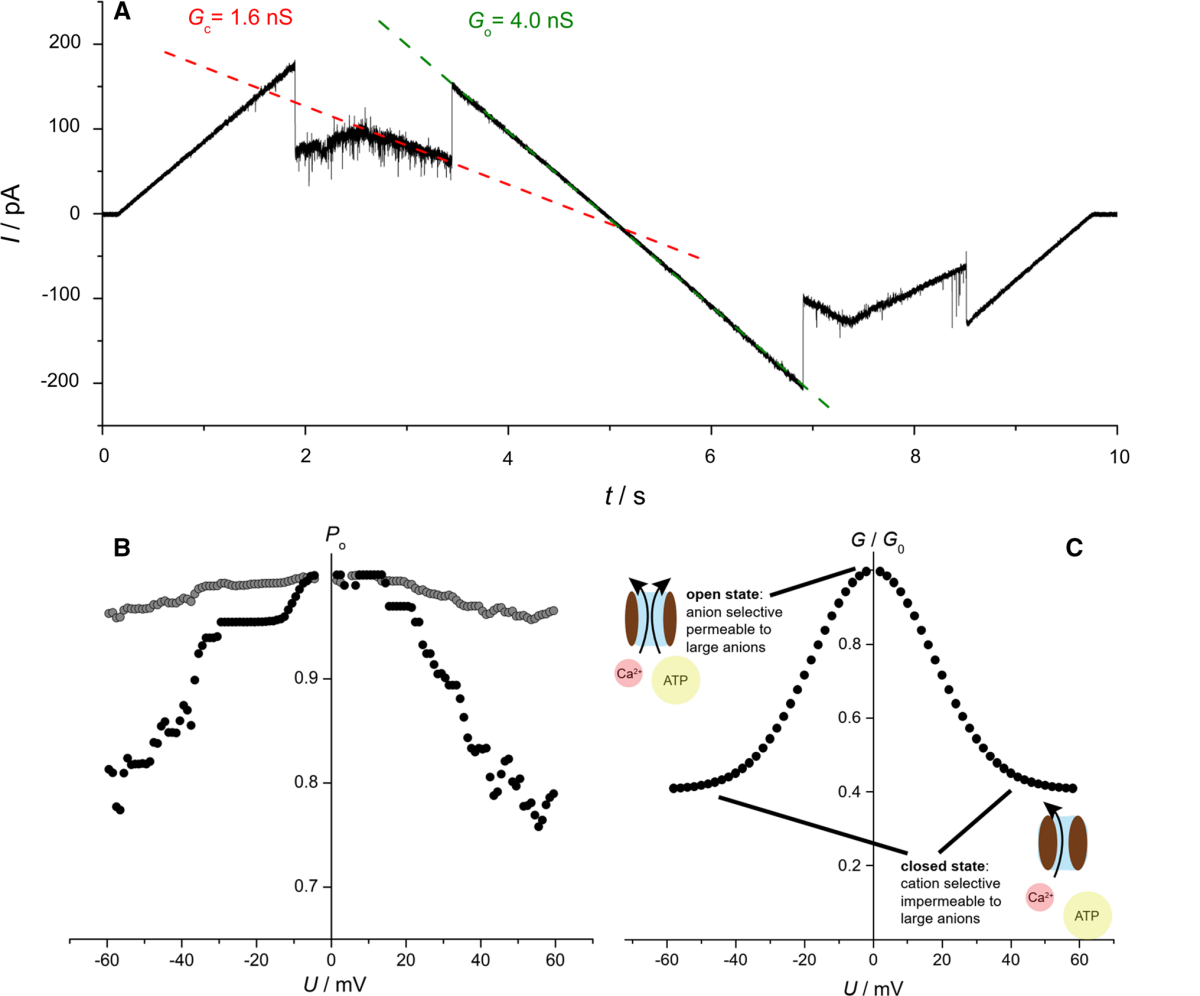
Characterization of VDAC by electrophysiology in a planar lipid bilayer (Port-a-Patch, Nanion) obtained by spreading of a protein-containing giant unilamellar vesicle. **a** Representative current trace of a single hVDAC1 channel. The slopes show the difference between the open state conductance of *G*_o_ = 4.0 nS (green dotted line) and a ‘closed’ state conductance of *G*_c_ = 1.6 nS (red dotted line). **b** Open probability *P*_o_ of a single hVDAC1 channel. If the open probability is calculated from all consecutively recorded voltage waves (*N* = 185), the grey curve is obtained. Considering only those traces, where the channel re-opened (18% of all traces) lowers the open probability at higher potentials (black dots). The channel was reconstituted into a solvent-free membrane composed of DPhPC/cholesterol (9:1) bathed in 1 M KCl, 1 mM CaCl_2_, 5 mM HEPES, pH 7.4. 100 MHz symmetrical triangular voltage waves with amplitudes of ± 60 mV were applied. **c** Idealized voltage dependence of the steady state conductance of the VDAC channel (*G*) relative to the conductance at low voltage (*G*_0_)

Here we describe only the solid-state NMR methods we used to obtain new experimental data, shown in Figs. 4 and 6. The methods used for electrophysiology experiments (Fig. 1a, b), are briefly summarized in the figure caption, and further details can be found in (Briones et al. 2016). The materials and methods for all other, previously published results can be found in the cited sources.

^2^H, ^13^C, ^15^N-labeled and fully back-exchanged hVDAC1 was expressed, refolded, and purified using established protocols (Bayrhuber et al. 2008; Hiller et al. 2008; Malia and Wagner 2007). We used the E73V mutation and also substituted cysteine residues, as C127A and C232S for NMR measurements. Preparation of 2D crystals was carried out as described in reference (Eddy et al. 2012). Porcine brain extract was purchased from Avanti, and for reconstitution a lipid to protein ratio (*n*/*n*) of 26:1 was used. To test cholesterol binding, a 26:1 lipid to protein ratio (*n*/*n*) 2D crystalline sample was prepared with d_54_-DMPC, and cholesterol was added at a 1:5 VDAC to cholesterol molar ratio (*n*/*n*).

All magic-angle spinning (MAS) spectra were recorded on a narrow-bore Bruker 800 MHz spectrometer in a 3-channel probe (^1^H, ^13^C, ^15^N) at 55 kHz spinning frequency in a 1.3 mm rotor, with the VT gas flow was set to 250 K. Typical pulse lengths of 2.5 μs (^1^H), 3.1 μs (^15^N) and 4 μs (^13^C) were used for all measurements. Heteronuclear magnetization transfers were implemented using cross-polarization. During evolution periods, 12.5 kHz TPPM decoupling (Bennett et al. 1995) was used on ^1^H, and 10 kHz WALTZ-16 decoupling (Shaka et al. 1983a, b) on both heteronuclei. Water suppression was achieved with the MISSISSIPPI (Zhou and Rienstra 2008) scheme applied at 13.75 kHz. The 4D HhCANH experiment (Najbauer et al. 2019) was recorded using 3.55% non-uniform sampling, with the NOE mixing time set to 75 ms. The spectra were referenced to 4.7 ppm water chemical shift, assuming a sample temperature of 30 °C based on external calibration. Typical acquisition times were 10 ms in the direct ^1^H dimension, 20 ms on ^15^N, 10 ms on ^13^C, and 5.3 ms in the indirect ^1^H dimension. The data were processed in Topspin 3.5pl7 and analyzed in Sparky.

### VDAC electrophysiology

VDAC derives its name from its characteristics displayed in reconstituted form under applied voltage, exhibiting a voltage-dependent conductance and selectivity (Schein et al. 1976). Such measurements are performed in 1,2-Didiphytanoyl-*sn*-glycero-3-phosphocholine (DPhPC) and cholesterol-based planar lipid bilayers (Port-a-patch, Nanion). At voltages below ± 20 mV, the channels are in an anion selective (2:1 for equally mobile Cl¯/K^+^) open state, exhibiting an average conductance of 4 nS in 1 M KCl (Benz 1994). In this conductive state, VDAC is also permeable to large anions, including ATP. At increasing membrane potentials of >  ± 40 mV (Schein et al. 1976), conductance drops to about half (Benz 1994), accompanied by a potential decrease in diameter (Colombini et al. 1987; Mannella and Guo 1990). In this less conductive state, the channel remains permeable to small ions, with a moderate preference towards small cations (Benz 1994; Benz and Brdiczka 1992; Hodge and Colombini 1997), but no longer permeable to ATP (Rostovtseva and Colombini 1996, 1997). Although it is widely accepted that VDAC’s open state is anion-selective, a cation-selective open state of the channel has also been observed (Pavlov et al. 2005).

There are two ways to illustrate the voltage dependency of VDAC in single channel recordings. First, the open probability *P*_o_ of a single VDAC as a function of the applied transmembrane potential *U* can be calculated from the corresponding current traces (Fig. 1a). As shown in Fig. 1b, the change in open probability as a function of applied voltage is very minor (gray curve), if all current traces are taken into account (Briones et al. 2016). If only those current traces are included, where the channel closes and re-opens within one voltage wave, as proposed by others (Teijido et al. 2012), the open probability drops below 80% at *U* =  ± 60 mV (black curve). Second, the steady state conductance of the VDAC channel (*G*) relative to its conductance at low voltage (*G*_0_) can be plotted (Fig. 1c) (Noskov et al. 2016). While the open channel shows a conductance of *G*_0_ = 4 nS, the conductance decreases in a stepwise fashion to values below 3 nS, referred to as ‘closed’ states, which are typically around 2 nS (Mertins et al. 2012), if the transmembrane potential exceeds ± 30 mV. In the example shown in Fig. 1a, *G*/*G*_0_ = 1.6 nS/4.0 nS = 0.4. The exact shape of the *G*/*G*_0_ curve is dependent upon details of the preparation, including lipid and buffer conditions.

**Fig. 2:**
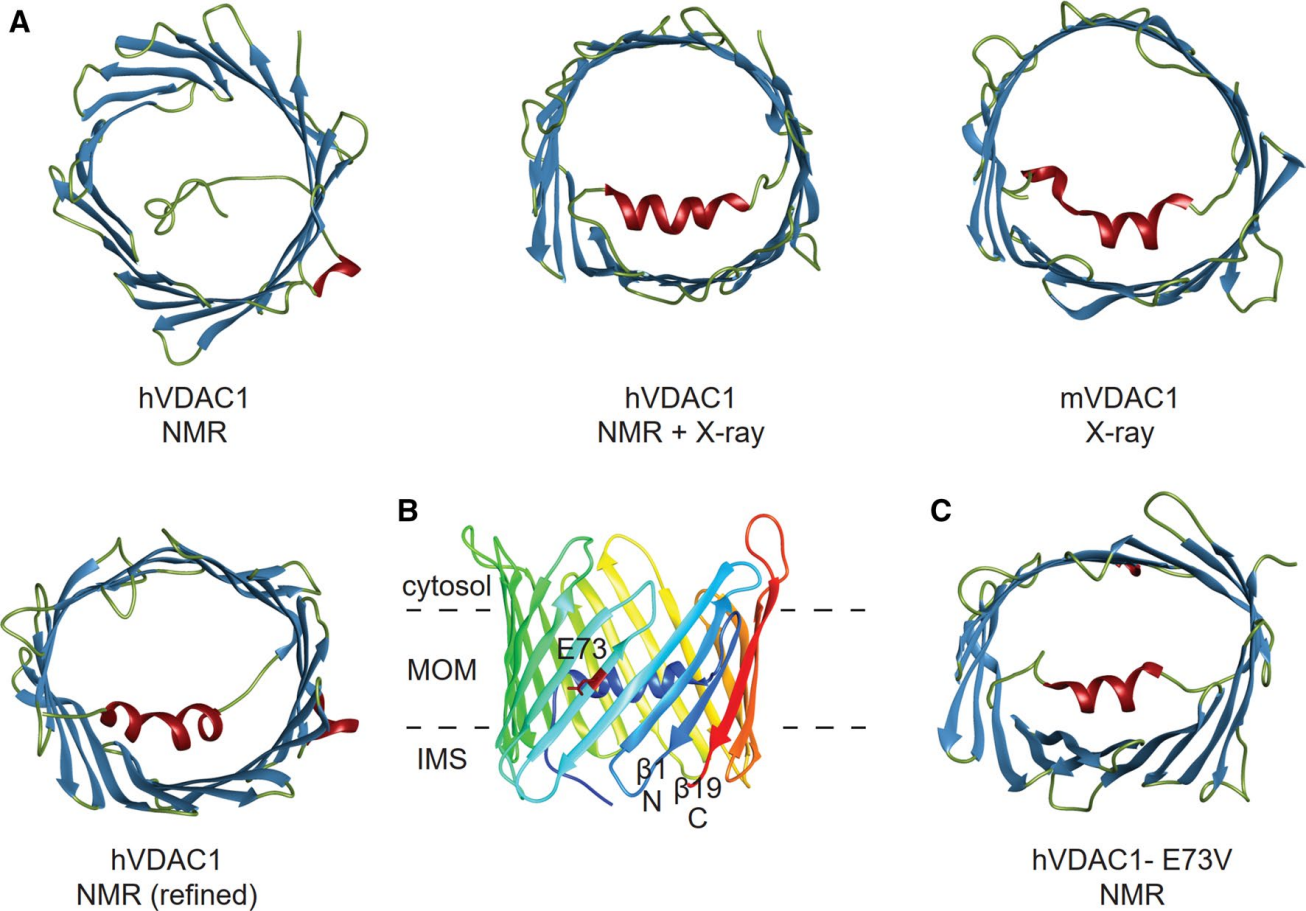
The structure of VDAC1. **a** The structure of human VDAC1 as determined by NMR spectroscopy (PDB code: 2k4t) (Hiller et al. 2008), a combination of NMR spectroscopy and X-ray crystallography (PDB code: 2jk4) (Bayrhuber et al. 2008), and the structure of mouse VDAC1 as determined by X-ray crystallography (PDB code:3emn) (Ujwal et al. 2008). A refined NMR structure of hVDAC1 is shown bottom left (PDB code: 6tiq) (Bohm et al. 2020). The structures are colored according to secondary structural elements (helix: red, β-strand: blue, unstructured region: olive). For the NMR structures the lowest energy structure of the ensemble was selected. **b** The structure of hVDAC in side view (PDB code: 2jk4). The dashed lines indicate the insertion of the channel into the mitochondrial outer membrane. The structure is colored from blue at the N-terminus to red at the C-terminus. The parallel orientation of the first (β1, blue) and last (β19, red) β-strands is clearly visible. The charged sidechain of E73 pointing into the membrane is colored in red. **c** High resolution structure of E73V-hVDAC1 as determined by NMR spectroscopy (PDB code: 5jdp) (Jaremko et al. 2016). The structure is colored according to its secondary structural elements, as described in (**a**). Of the structural ensemble, the structure with the lowest energy has been selected for display

VDAC channels from distantly related eukaryotes have remarkably conserved biophysical properties, highlighting their universal importance (Blachly-Dyson and Forte 2001; Saccone et al. 2003). In mammals, VDAC is expressed in three isoforms. Interestingly, it is widely accepted that evolutionarily VDAC1 is the youngest of the three isoforms, VDAC3 having emerged first (Saccone et al. 2003). Both VDAC1 (Schleiff et al. 1999; Xu and Colombini 1996) and VDAC2 insert into membranes readily, are anion selective, and have similar voltage dependence (Menzel et al. 2009), while VDAC3 seems to have a low propensity for membrane insertion and does not gate well, even at high membrane potentials of ± 80 mV (Xu et al. 1999). This suggests that VDAC3 might have a function other than altering the ion- and metabolite flow through the OMM. The three isoforms of mammalian VDAC are expressed ubiquitously, although at varying levels in different tissues, for example both VDAC2 and 3 have high expression levels in sperm outer dense fibers (Hinsch et al. 2004). Of the three isoforms, VDAC1 is by far the most abundant (Messina et al. 2012), and is also best described in literature.

### VDAC structure

Initial predictions of VDAC structure based on biochemical data from mutagenesis studies, and antibody assays, as well as computational approaches all pointed to the formation of a β-barrel, but with varying number of β-strands. Estimates ranged from 12 strands (Blachly-Dyson et al. 1989, 1990), 13 (Song et al. 1998b), 16 (Casadio et al. 2002; Depinto et al. 1991; Rauch and Moran 1994), 18 (Al Bitar et al. 2003), and up to the value eventually determined in the NMR and X-ray structures, of 19 (Forte et al. 1987) strands. Though initially debated (Forte et al. 1987), existence of an amphipathic N-terminal α-helix was widely accepted and later proven experimentally (De Pinto et al. 2007) in both detergents and lipids (Shanmugavadivu et al. 2007), however it remained unclear whether the helix forms part of the barrel wall (Blachly-Dyson et al. 1989, 1990), or is exposed to the water phase (De Pinto and Palmieri 1992), perhaps extending away from the pore lumen (Guo et al. 1995).

Figure 2 shows ribbon representations of atomic resolution VDAC structures rendered with Chimera (Pettersen et al. 2004)*.* The first of these structures were reported in 2008 using VDAC preparations in a detergent environment, using NMR spectroscopy (Hiller et al. 2008), the second also from micelles using a combination of NMR and X-ray crystallography (Bayrhuber et al. 2008), and the third from bicelles instead of micelles using X-ray crystallography alone (Ujwal et al. 2008). All three structures unequivocally showed a 19-stranded β-barrel, with the N-terminal segment positioned inside the pore. The latter two structures resolved the N-terminal helix positioned in contact with the barrel wall. The odd number of β-strands in VDAC was quite surprising, the parallel beta sheet interaction between the first and the last strands (β1 and β19) were never before observed in other integral membrane proteins. Astonishingly, a similar topology with 19 β-strands and a helix in the opening of the pore was recently resolved in TOM40, also located in the MOM (Araiso et al. 2019; Tucker and Park 2019). Slight differences in the structure occur for the N-terminal helix resolved in the NMR-X-ray hybrid structure (Bayrhuber et al. 2008) and in the structure solved exclusively by X-ray crystallography (Ujwal et al. 2008). Bayrhuber et al. found one helix spanning residues 7–17, while Ujwal et al. resolved two helices, comprised of residues 6–9 and 12–20, with L10 and G11 forming a kink between the two. In both cases, the helical N-terminus runs along the barrel wall, its position stabilized by hydrogen bonds between the N-terminus and the barrel. The residues forming the barrel have an alternating pattern of hydrophilic and hydrophobic sidechains pointing into the aqueous pore lumen and the hydrophobic environment, respectively. On the exterior, this pattern is broken only by residue E73 in β4, for which the negatively charged glutamate sidechain faces the hydrophobic environment.

An NMR study of E73V hVDAC1 used relaxation data to determine a high resolution structure of the protein (Jaremko et al. 2016). This structure resolved two helices in the N-terminus, unambiguously showing that it was not elevated N-terminal dynamics, but rather a lack of resolution that resulted in the discrepancy of the conformation of the N-terminus in the three initial structures from 2008. Interestingly, the barrel showed a distinct elliptic deformation that was not observed in the crystal structure of the mouse variant in bicelles. It is unclear whether the E73V mutation stabilizes an existing conformation of wild-type VDAC, resulting in a predominantly elliptic barrel shape, or it is the pressure exerted by micelles that deforms the barrel, while bicelles do not exert this pressure.

The structure of VDAC2 is very similar to that of VDAC1, as shown by X-ray crystallography and solid-state NMR spectroscopy (Gattin et al. 2015; Schredelseker et al. 2014), including nearly identical dynamic behavior and conformational homogeneity (Eddy et al. 2019). Although to date, VDAC3 has not been structurally characterized, the sample preparation and spectroscopic methods described by Eddy et al. will likely be applicable to this isoform as well (Eddy et al. 2019).

### Structural studies of VDAC in a lipid bilayer

Studying VDAC in a lipid bilayer as opposed to micelles or bicelles has always been a rather challenging undertaking. Extensive efforts to determine the structure using tomography resulted in only 8.2 Å resolution of the pore (Dolder et al. 1999). The protein is also challenging to address using solid-state NMR due to the sheer size of the protein which results in a requirement for very high sample homogeneity. VDAC alone is still too small for high resolution characterization with single particle cryo-electron microscopy.

In liposome preparations, NMR data (Schneider et al. 2010) indicated that the N-terminus of human VDAC1 (hVDAC1) exists in a rigid and well-defined structure. Additionally, (^13^C,^13^C) dipolar order parameters (S_CC_) were measured for residues in the N-terminus as well as in other parts of the molecule using double-quantum (2Q) spectroscopy, a technique that is sensitive to dynamics on the pico- to millisecond timescale (Zachariae et al. 2012). Due to the considerable size of hVDAC1 and the large number of residues in β-sheet conformation, spectral overlap precluded identification of residue-specific order parameters for a large part of the molecule. To estimate overall mobility in the β-barrel, overlapping signals were analyzed to determine average order parameters. The data showed that, globally, the N-terminus is clearly not more flexible than the β-barrel on a sub-ms timescale, as might be expected if the N-terminus were primed to move under applied voltage (see voltage gating discussion below). Peak broadening or doubling, which would have indicated dynamics on slower timescales, was also not observed, further confirming the well-defined structure of the hVDAC1 N-terminus.

In the above liposome preparations, with the exception of three residues, the spectral quality was insufficient to resolve and assign barrel resonances. This emphasizes an extremely stringent requirement for preparation of homogeneous VDAC samples that may be influenced by VDAC’s high propensity for oligomerization (Goncalves et al. 2007; Hoogenboom et al. 2007), resulting in a microscopically inhomogeneous sample, despite high sample purity.

Under certain conditions, in the presence of lipids, membrane proteins may form multilamellar crystalline arrays (2D crystals). Importantly, 2D crystals allow investigation of proteins in a native-like lipid bilayer environment, where they often retain full functionality, while the high microscopic order in these preparations has long been exploited by electron microscopy (Jap et al. 1990; Kuhlbrandt and Wang 1991; Unger et al. 1997; Walz and Grigorieff 1998), atomic force microscopy (Stahlberg et al. 2001), and solid-state NMR spectroscopy (Hiller et al. 2005; Lewis et al. 1985; Shastri et al. 2007) to gain atomic-level structural information.

**Fig. 3.**
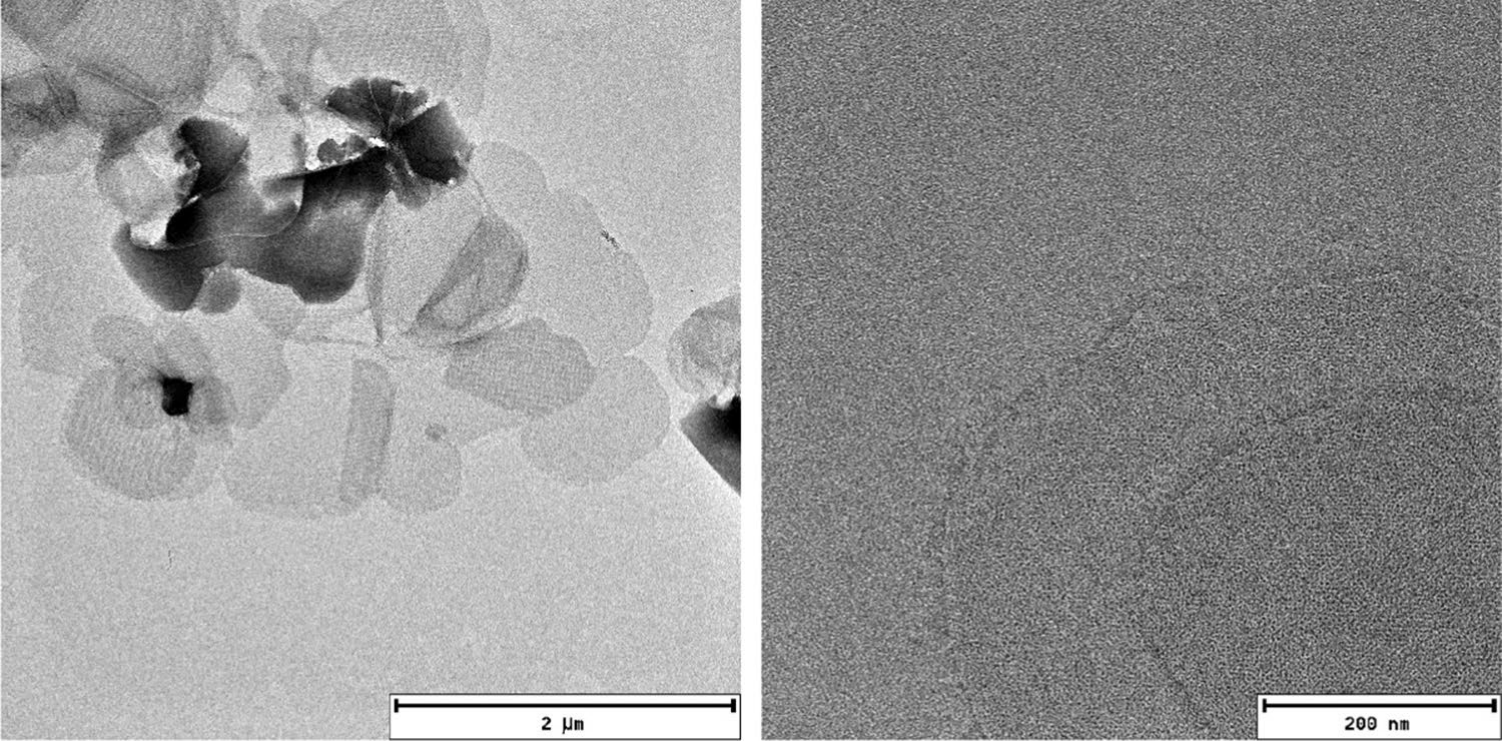
2D crystals of VDAC. Negative stain electron microscopy images of VDAC 2D crystals at different resolutions. Horizontal bars indicate relative sizes. The lamellar structure of the crystals is clearly visible on both images. On the right, VDAC channels can be seen as small black dots

**Fig. 4:**
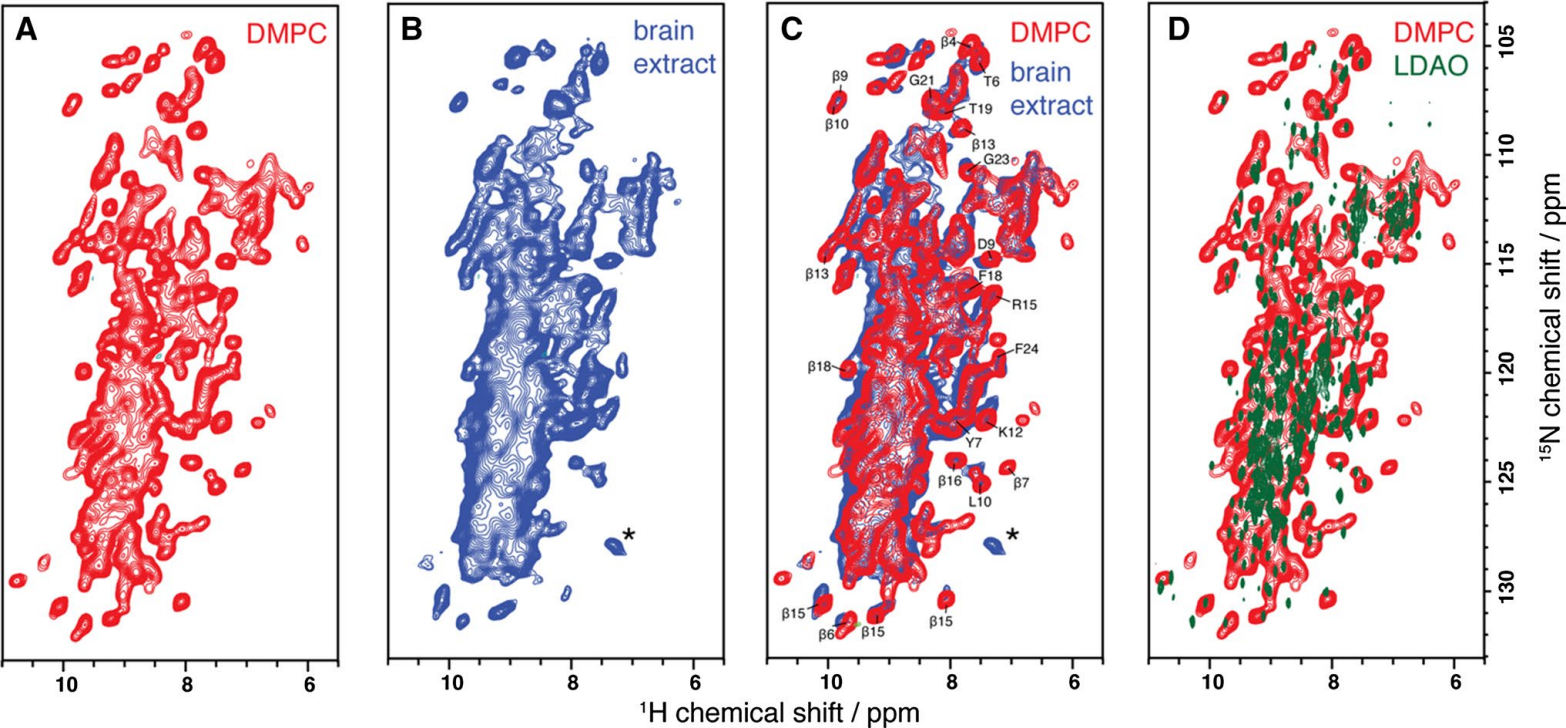
hNH correlation spectra of E73V hVDAC1 reconstituted in **a** DMPC and **b** brain extract. **c** shows the overlay of spectra recorded in DMPC and brain extract, as well as assignments for isolated peaks from the α-helix, the kink between the helix and the barrel, and from several β-sheets. Due to the lower spectral width of the spectrum recorded in brain extract lipids, an aliased sidechain appears, marked with an asterisk. In **d**, the spectrum is compared with the TROSY spectrum of E73V hVDAC1 in LDAO micelles, as used for WT protein in Hiller et al. 2008 and Bayrhuber et al. 2008. Cysteine residues were substituted as C127A and C232S

At relatively low lipid-to-protein ratios, VDAC also forms 2D crystals (Dolder et al. 1999) of fully functional channels (Fig. 3) (Eddy et al. 2012). In contrast to VDAC1 in LDAO micelles, the presence of cholesterol or the detergent Triton X-100 is not required for the formation of the crystalline arrays, or for the channel to be fully functional, as determined with electrophysiology. Furthermore, the structure of the protein remains unchanged upon 2D crystal formation in various lipids (Eddy et al. 2012). We found that this also holds true under a near native lipid composition using a total lipid extract from porcine brain. The spectrum is shown in Fig. 4, and shows only minor perturbations as compared with the single component phosphocholine lipid DMPC. In particular, the peak positions for helical residues labeled in Fig. 4c are nearly unchanged. The minor chemical shift changes observed for beta strand residues are therefore most likely caused by local interaction with the different lipid components of the brain extract. Chemical shifts measured in LDAO micelles (Fig. 4d) and DMPC lipid bilayers matched well overall, and indicate a highly similar fold in both environments. The 2D crystalline preparations yield excellent quality solid-state NMR spectra (heteronuclear linewidths < 0.5 ppm), and—perhaps due to a reduction of local mobility by tight packing—also facilitate the assignment of loop regions. Using carbon detection and several samples with different isotopic labeling, it was possible to assign 88 residues, including most of the α-helix, as well as residues from β-strands 5,6,9,13,18, and 19 (Eddy et al. 2015b). The use of both uniformly ^13^C, ^15^N-labeled VDAC as well as several inverse labeled samples was key to reduce ambiguity for assignment of beta strands. The assignments shown in Fig. 4 were determined using a suite of proton detected 3D spectra (Barbet-Massin et al. 2014).

**Fig. 5:**
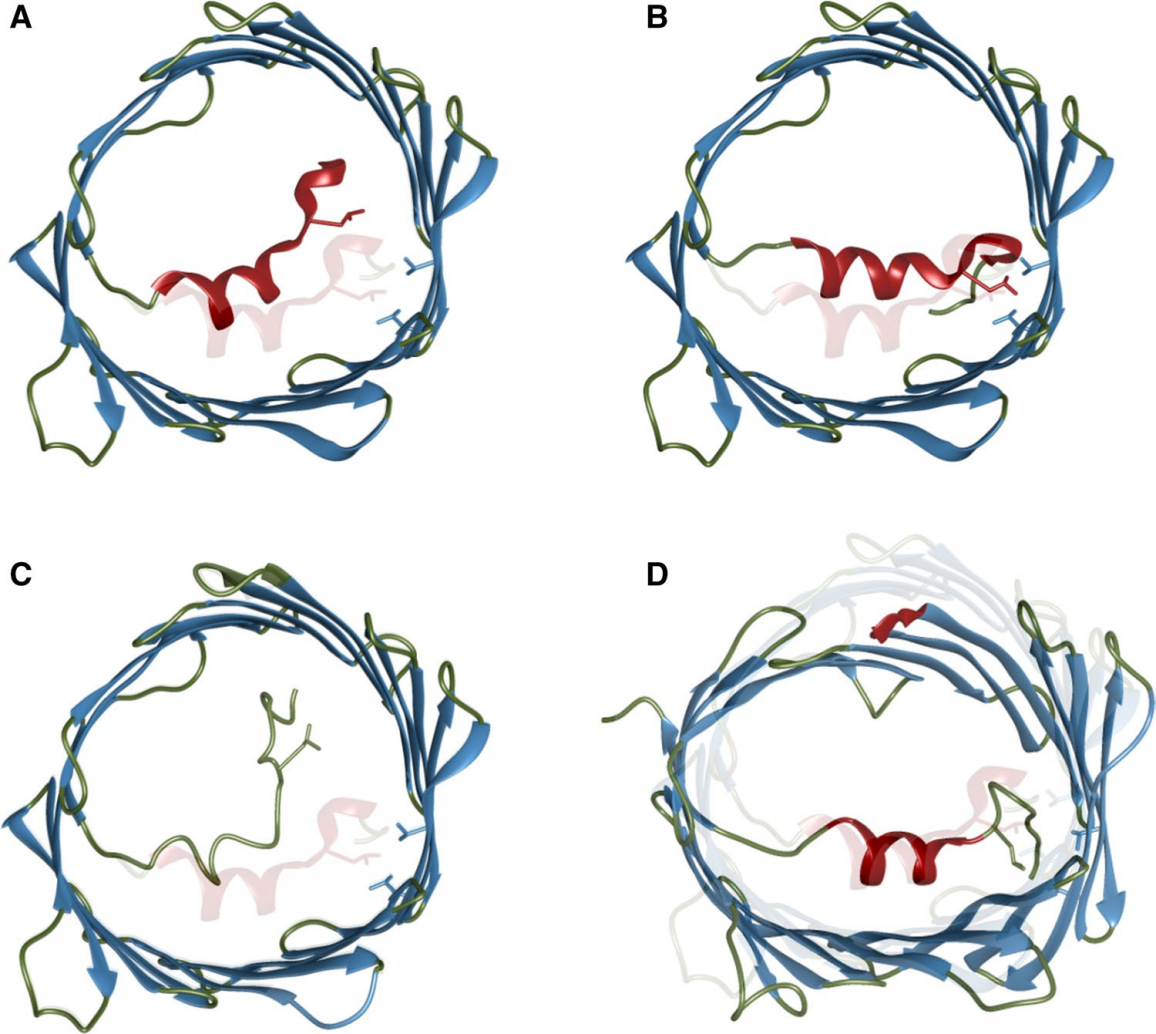
Models for VDAC’s voltage gating mechanism. **a** Voltage gating by movement of the helix to the center of the pore (Ujwal et al. 2008). **b**) Voltage gating through dislodging of the α2-helix from the barrel wall (Hiller and Wagner 2009). **c** Unwinding of the helix upon gating (Zachariae et al. 2012). **d** Voltage gating upon elliptic deformation the barrel without dislodging of the helix (Villinger et al. 2010; Zachariae et al. 2012). The depictions were generated by manual modification of the VDAC structures deposited under pdb ID 3emn, 5jdp and 2k4t

The structure of both high and low conductance states of VDAC has long been controversial. It has been argued that while published structures of the protein show the existence of a 19-stranded β-barrel, functional studies point to a barrel formed from 13 β-strands and an α-helix (Colombini 2012). Secondary chemical shifts calculated from assignments in a lipid bilayer can give some insight into the secondary structure in its functional open state. To date, this information confirms the presence of several beta sheets matching those of the 19-stranded structure, yet information is incomplete due to the lack of solid-state NMR assignments for many strands. With the exception of a long-range contact between A14 C_β_ in the N-terminal helix and S193 C_β_ (Eddy et al. 2015a), there is no information available on the 3D structure of VDAC in a lipid bilayer.

To mimic a lipid bilayer environment in solution, preparation of VDAC in nanodiscs has also been established that yield high resolution NMR spectra (Raschle et al. 2009), and later further improved by employing covalently circularized nanodiscs (Nasr et al. 2017). While these samples yield excellent quality 2D spectra, recording higher dimensionality experiments necessary for sequential assignment may be difficult due to fast transverse relaxation rates, resulting in low efficiency magnetization transfer, or a requirement for elevated temperatures.

### VDAC’s gating mechanism

The mechanism of VDAC’s voltage gating is not yet understood, though several models exist. Early electrophysiology studies on VDAC reconstituted in lipid bilayers suggested changes in the channel diameter upon gating, from ~ 3 nm to 1.8 nm (Colombini et al. 1987; Zimmerberg and Parsegian 1986). A series of mutagenesis and biotinylation studies identified several residues throughout the protein sequence to influence gating behavior (Blachly-Dyson et al. 1990; Song et al. 1998b; Thomas et al. 1993), and this observation led to the proposal of a gating model, in which VDAC gating is accomplished by a complex rearrangement of the protein involving the movement of a large, positively charged “voltage sensor” region of the protein out of the membrane, resulting in a smaller pore (Song et al. 1998a; Thomas et al. 1993).

After the publication of VDAC’s high resolution structure in 2008 (Fig. 2), several models for gating were proposed based on the fact that the N-terminal α-helix is located inside the pore, by the barrel wall, at a key position to regulate gating. A possibility suggested by Hiller et al. was that the N-terminus adopts different conformations upon gating (Hiller et al. 2008), possibly including unwinding of the helix (Fig. 5c) (Zachariae et al. 2012). Another suggestion was that upon gating the entire helix moves to the center of the channel, thus obstructing the flux of ions and metabolites (Fig. 5a). In this case modulators, such as NADH would close the channel by binding to the interaction site between the helix and the barrel wall, disrupting the hydrogen bonding pattern and dislodging the helix (Ujwal et al. 2008). A recent study has shown however, that reduction of channel conductance upon NADH binding occurs through NADH sterically blocking the pore, during which the helix conformation is essentially unchanged. This is in contrast to the mechanism of channel closure upon voltage gating, the basis for which is increased mobility of the N-terminus, in particular the α2 helix, as dynamics and electrophysiology measurements on cross-linked VDAC mutants suggest (Bohm et al. 2020).

These electrophysiology studies investigating cross-linked VDAC mutants yield further insight into the role of the N-terminus. Affixing the very end of the N-terminus results in loss of symmetric voltage response (V3C-K119C), while cross-linking the α2 helix to the barrel wall (A14C-S193C) locks the channel into a permanently open state potentially providing support for the gating mechanism illustrated in Fig. 5b (Mertins et al. 2012). Notably, a cross-link between L10 in the kink between the two helices and the barrel wall (L10C-A170C) does not prevent the channel from closing under applied voltage, which suggests that the N-terminus does not completely move away from the barrel wall upon gating (Teijido et al. 2012). This excludes the gating model illustrated in Fig. 5a.

Electrophysiology studies have also shown that the voltage-dependence of VDAC gating is modulated by the lipid composition of the surrounding membrane (Rostovtseva et al. 2006). Because the N-terminal α-helix is positioned inside the pore and does not directly contact the membrane, additional regions within the β-barrel are likely to contribute to the voltage sensitivity of VDAC. A combination of NMR dynamics studies and MD simulations performed by Villinger et al. showed that the N-terminal six β-strands, which contain several residues important for gating undergo μs-ms motion (Villinger et al. 2010). The motions result in deformation of the barrel including the elliptical barrel of the E73V mutant (Fig. 5d), which can influence the diffusion of small ions and metabolites through the channel. MD simulations in combination with solid-state NMR spectroscopy further showed the occurrence of barrel deformations and concomitantly a decrease of channel conductance (Briones et al. 2016).

These barrel deformations, leading almost to the faltering of the barrel, are most drastically observed upon removal of the N-terminal helix (Schneider et al. 2010). MD simulations predict significant displacement of charged helical residues in the N-terminus, especially in the K12-K20 region, as well as residues in β-sheets 1, 3, 7 and residues 104–107 and 266–268 located in cytoplasmic loops (Briones et al. 2016). At least partial detachment of the α-helix from the barrel wall might therefore result in faltering of the barrel. Displacement of barrel residues upon voltage gating was further supported by surface-enhanced infrared absorption and electrochemical impedance spectroscopy (Kozuch et al. 2014). The combined data support a gating model in which transport of small molecules and metabolites is regulated via elliptic deformations of the VDAC β-barrel coupled to detachment of the C-terminal part of the N-terminal α-helix.

There are still many controversies regarding the mechanism of gating. While a recent publication finds E73 is not involved in the gating (Queralt-Martin et al. 2019), NMR spectroscopy finds a clear reduction of barrel motion in locations that are involved in the barrel faltering, and older electrophysiological measurements on the E73Q mutant confirm reduced voltage dependence (Zaid et al. 2005). Reconciling the partially contradictory information regarding the gating behavior of VDAC, and understanding the role of the N-terminus will likely require structural studies in a native-like detergent-free environment, where detergents cannot disrupt helix-barrel contacts, as well as further MD simulations.

### VDAC interactions

VDAC regulates cellular metabolism and the exchange of ATP, ADP, phosphate, Ca^2+^ and various small molecules between mitochondria and the cytoplasm not only through its gating behavior but also through its interactions with a large variety of molecules, making it an important checkpoint in controlling various cellular processes.

Mitochondrial function and ATP production is largely dependent on mitochondrial Ca^2+^ concentration (Gunter and Sheu 2009). As the primary avenue for the transport of Ca^2+^ and ATP through the mitochondrial outer membrane, VDAC is a key player in controlling mitochondrial Ca^2+^- homeostasis and cellular function (Shoshan-Barmatz et al. 2018a). VDAC binds (Gincel et al. 2001) and permeates Ca^2+^ in both its open and closed states (Tan and Colombini 2007), and this binding has been shown to influence molecular plasticity, possibly altering the channel’s gating behavior (Ge et al. 2016). ATP molecules reversibly bind to VDAC as they pass through the channel (Florke et al. 1994), and a low-affinity interaction site on the N-terminal helix (Yehezkel et al. 2007) and the adjacent β-barrel residues (Villinger et al. 2014), as well as the mechanism for permeation have been identified (Choudhary et al. 2014). Other nucleotides, including GTP and UTP share a common binding site with ATP, and partially overlap with *β*-NADH binding sites previously identified by NMR spectroscopy (Hiller et al. 2008; Villinger et al. 2014).

**Fig. 6:**
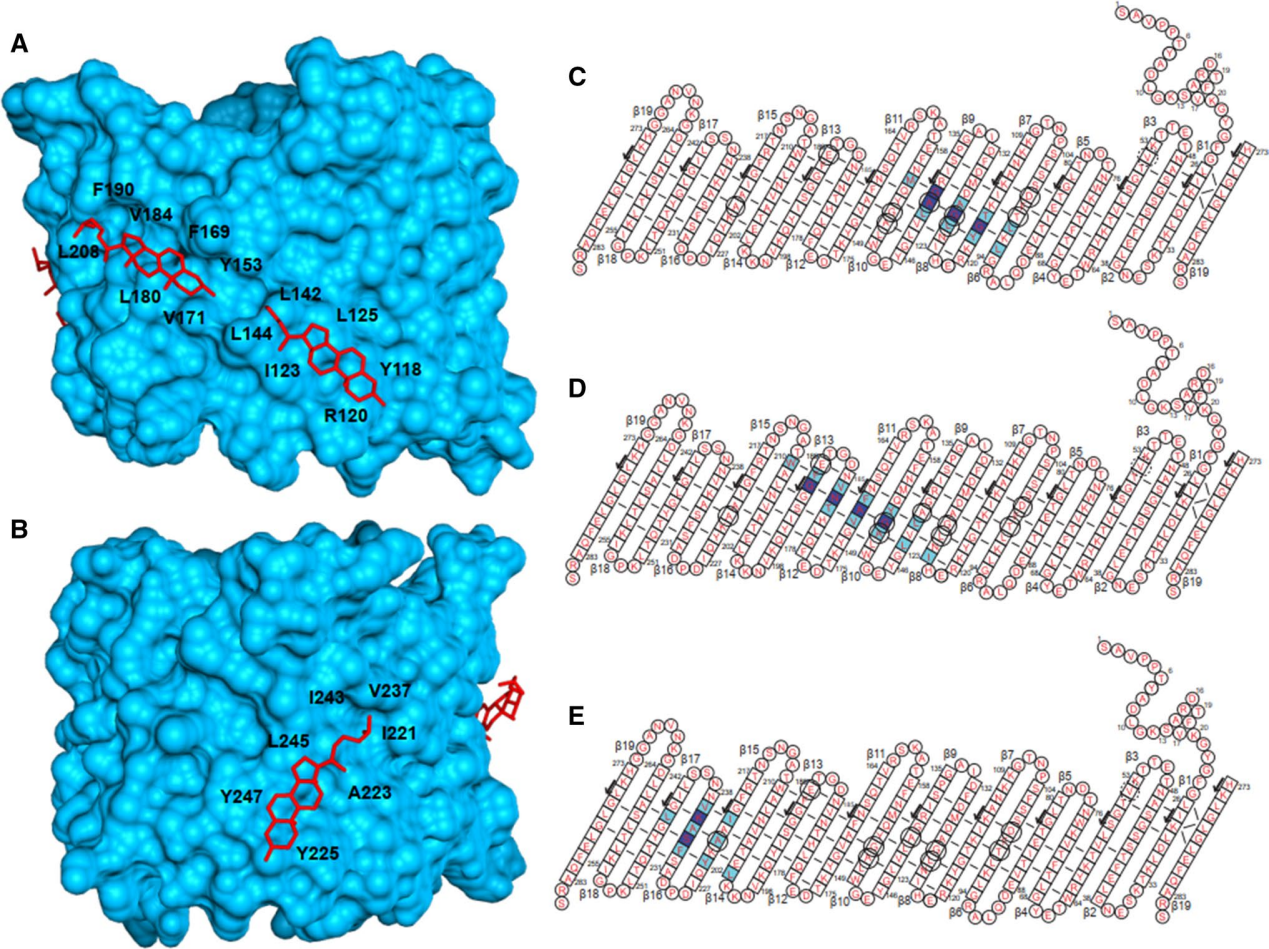
Interaction of E73V hVDAC1 with cholesterol. **a** and **b** show the three cholesterol binding sites of VDAC identified by solid-state NMR spectroscopy. These are a subset of the five binding sites predicted by docking (Weiser et al. 2014). The hydrophobic sidechains forming the grooves on the side of the barrel are labeled. **c**, **d**, and **e** show the cholesterol aliphatic chain– protein amide contacts observed by solid-state NMR circled in black. Backbone and sidechain contacts predicted by docking are colored in dark and light blue, respectively. Cysteine residues were substituted as C127A and C232S

VDAC also binds a variety of other small molecules, whose function is still not fully understood. Cholesterol has been shown to co-purifiy with VDAC at a 5:1 ratio (De Pinto et al. 1989), and cholesterol binding has been suggested to modulate the channel’s behavior and to be necessary for VDAC to achieve full function (Mlayeh et al. 2010; Popp et al. 1995), though other evidence suggests it does not seem to influence basic channel properties (Eddy et al. 2012; Queralt-Martin et al. 2019). At least two potential cholesterol binding sites in detergent micelles have been identified using NMR spectroscopy (Hiller et al. 2008), and a docking study found these two patches to comprise five distinct binding sites (Weiser et al. 2014). Using photo-affinity labeling and mass spectrometry, cholesterol was found to bind to four of these sites, as well as a site near E73, which could potentially have implications for channel mobility, and thus, gating. Using solid-state NMR spectroscopy to observe direct contacts between cholesterol sidechains and the protein backbone, we could identify three of the binding sites predicted through docking. The approximate placement of cholesterol in these binding modes is shown in Fig. 6a, b, and contacts with cholesterol are indicated as circled residues in Fig. 6c, e. We used a direct magnetization transfer to observe the cholesterol protons, and both protein and DMPC lipid components were deuterated to suppress aliphatic signals (Najbauer et al. 2019). Good agreement is seen with the predicted contacts based on molecular docking and shaded in blue in Fig. 6c, e for 3 cholesterol binding sites. Other sterols, such as the neurosteroid allopregnanolone also largely share these binding sites (Cheng et al. 2019).

Cholesterol-binding CRAC (Cholesterol Recognition/interaction Amino acid Consensus sequence) and CRAC motifs have so far only been identified in transmembrane helices (Fantini and Barrantes 2013). The CRAC motif is defined by a (L/V)-X_1−5_-(Y)-X_1−5_-(K/R) pattern of residues, whereas the CARC motif is inverted, and may also include phenylalanine at the central position: (K/R)-X_1−5_-(Y/F)-X_1−5_-(L/V). We observe that the grooves on the barrel wall accommodating the bound cholesterol are formed by the hydrophobic residues also found in CRAC and CARC motifs. Positively charged arginine (R120, R139) and lysine (K236) residues can be found in the vicinity of the binding sites, but two of them, R139 and K236 face towards the pore lumen, excluding the possibility of interaction with the bound cholesterol. This leaves only one site with all three characteristics of the CRAC motif. We thus find that while for VDAC’s β-barrel the presence of a positive charge is not required, the interacting hydrophobic residues (Y and L found in all binding sites) are the same as observed for the CRAC motif.

VDAC’s opening/closing is an important checkpoint in cellular metabolism, and is regulated through a large variety of protein–protein interactions as reviewed recently (Caterino et al. 2017). VDAC exhibits pro-apoptotic activity by interacting with several proteins from the apoptosis-related Bcl2 protein family. Through its interaction with Bax and Bak, VDAC has been suggested to participate in the release of cytochrome *c* into the cytosol and the activation of the apoptotic cascade (Shimizu et al. 2000; Tsujimoto and Shimizu 2000), while the anti-apoptotic Bcl-x_L_ protein closes the pore by direct binding (Shimizu et al. 1999). Hexokinase 1 binding to VDAC may suppress apoptosis by modulating VDAC activity and controlling the channel switching between off and on states (Caterino et al. 2017; Dubey et al. 2016). VDAC’s inhibition by tubulin could influence ATP trafficking, and even induce a switch, known as the Warburg effect, between oxidative phosphorylation and glycolysis in cancer cells (Maldonado et al. 2013).

VDAC is a promising drug target in the therapy of neurodegenerative and cardiovascular diseases associated with mitochondrial dysfunction, as well as cancer as recently reviewed (Magri et al. 2018). For cancer therapy, drug candidates rely mainly on inducing apoptosis by direct blockage of the channel or through promoting VDAC oligomerization, however many of these drug candidates struggle with lack of selectivity, difficult delivery or high toxicity. A particularly promising molecule that has entered clinical trials for leukemia (O'Brien et al. 2007) and breast cancer (Moulder et al. 2008) is the 18-mer phosphorothioate oligonucleotide G3139 (also known as Genasense or oblimersen). It was developed as an antisense oligonucleotide, complementary to the first six bases of the anti-apoptotic protein Bcl2- mRNA, however it has been shown to selectively bind to VDAC, blocking the channel (Lai et al. 2006; Tan et al. 2007). The mechanism of blockage is unknown, though the kinetics indicate at least a partial entry into the pore.

VDAC potentially forms important interactions with other integral membrane proteins collocated in the OMM. One such protein, the translocator protein (TSPO) has received attention as a marker for oxidative stress and inflammation. A series of PET ligands has been developed that bind TSPO with nanomolar affinity (Veenman et al. 2016), one of which was used to stabilize the structure for determination in detergent micelles (Jaremko et al. 2014). In lipid bilayers, it was possible to probe the influence of cholesterol and observe the equilibrium between monomer and dimeric forms of the protein (Jaipuria et al. 2017). Both VDAC (Shoshan-Barmatz et al. 2018b) and TSPO (Repalli 2014) are upregulated in the Alzheimer’s, a serious neurodegenerative disease. While there is evidence for VDAC-TSPO interaction in mouse models of Alzheimer’s (Oakley et al. 2006), to date the details of this interaction remain unknown. Due to the apparent importance of these two proteins in apoptosis, their interactions have been proposed as a potential drug target for future development (Veenman and Gavish 2006; Veenman et al. 2007).

## Conclusions

In summary, VDAC has been the focus of intense investigation in the last four decades via various biophysical techniques. This has led to an understanding of conductance properties that can now be related back to the channel’s open state structure without applied voltage as determined by NMR spectroscopy and X-ray crystallography. VDAC’s gating mechanism and its detailed structure in the closed state remain under investigation. We showed that the VDAC protein structure is stable in different membrane compositions, including conditions with cholesterol, which we localized via direct NMR measurements.
